# Effect of Ferulic Acid-Grafted-Chitosan Coating on the Quality of Pork during Refrigerated Storage

**DOI:** 10.3390/foods10061374

**Published:** 2021-06-14

**Authors:** Guotian Wang, Yunpeng Liu, Huimin Yong, Shuai Zong, Changhai Jin, Jun Liu

**Affiliations:** 1 Laboratory and Equipment Management Office, Yangzhou University, Yangzhou 225009, China; gtwang@yzu.edu.cn; 2 College of Food Science and Engineering, Yangzhou University, Yangzhou 225127, China; lyp1262021@126.com (Y.L.); yhm2021@126.com (H.Y.); shuaizong@yzu.edu.cn (S.Z.); chjin@yzu.edu.cn (C.J.)

**Keywords:** chitosan, ferulic acid, graft copolymer, edible coating, pork preservation

## Abstract

Pork is perishable due to oxidation and microbial spoilage. Edible coating based on biopolymers and phenolic compounds is an effective way to preserve the quality of pork. In this study, ferulic acid-grafted-CS (ferulic acid-g-CS) with strong antioxidant and antimicrobial activities was synthesized through a carbodiimide-mediated coupling reaction. The obtained ferulic acid-g-CS was used as an edible coating material for fresh pork. The effect of ferulic acid-g-CS coating on the quality of pork during storage was investigated at 4 °C for 8 days. As compared to the uncoated pork, pork coated with CS and ferulic acid-g-CS showed lower total viable counts, total volatile basic nitrogen values, pH values, thiobarbituric acid reactive substances, and drip losses. Besides, pork coated with CS and ferulic acid-g-CS presented more compact microstructures than the uncoated pork at the eighth day. Sensory evaluation assay showed pork coated with CS and ferulic acid-g-CS had better color, odor, and over acceptance in comparison with the uncoated pork. Ferulic acid-g-CS coating, due to its relatively higher antioxidant and antimicrobial activities compared to CS coating, had a better performance in refrigerated pork preservation. Ferulic acid-g-CS coating effectively extended the shelf life of refrigerated pork to 7 days. This study revealed ferulic acid-g-CS coating was a promising technology for refrigerated pork preservation.

## 1. Introduction

Pork is a popular meat product worldwide due to its pleasant flavor, juiciness and nutrition. However, fresh pork is susceptible to oxidation and microbial growth because it is rich in polyunsaturated fatty acids, lipids, and proteins [1]. Oxidation and microbial spoilage lead to discoloration, off-flavor, off-odor, nutrient loss, and deterioration in pork, which shortens the shelf life of pork [2]. Till now, a number of packaging technologies, such as active packaging, modified atmosphere packaging, vacuum packaging, and edible coating, have been used to prolong the shelf life of pork [3]. Among them, edible coating based on natural biopolymers (e.g., polysaccharides, proteins and lipids) has received great interests in pork preservation [4]. Edible coating is normally realized by brushing, dipping, or spraying biopolymer-based solutions on pork surface, which can function as a thin layer barrier to retard moisture loss, oxygen permeation and solute migration during pork storage [5,6]. Notably, the pork preservation efficiency is closely related to the formulation of edible coating. Therefore, researchers have focused on developing effective coating formulation for pork preservation [5]. 

Chitosan (CS) is an ideal biopolymer for the development of edible coating because CS is non-toxic and renewable [7]. Meanwhile, CS also possesses intrinsic antioxidant and antimicrobial activities, which are essential for active food packaging [8]. However, the limited antioxidant and antimicrobial activities of CS coating alone cannot satisfy effective pork preservation. Therefore, different natural active compounds (e.g., phenolic compounds, organic acids and essential oils) with strong antioxidant and antimicrobial activities have been incorporated into CS coating solutions [9,10,11,12,13]. Among different kinds of natural active compounds, phenolic compounds have received great attention due to their potent antioxidant and antimicrobial activities [14]. Nevertheless, the direct addition of phenolic compounds into CS coating presented several disadvantages, such as the low stability and rapid release of active compounds [15]. Therefore, it is essential to develop more stable coating systems based on CS and phenolic compounds.

In recent years, CS has been functionalized with phenolic compounds through different types of graft copolymerization reactions, such as carbodiimide-mediated coupling reaction, free radical-induced reaction and enzyme-catalyzed reaction [16]. Among these reactions, carbodiimide-mediated coupling reaction normally produces phenolic-grafted-CSs (phenolic-g-CSs) with the highest grafting efficiency [13]. Notably, the produced phenolic-g-CSs not only present stronger antioxidant and antimicrobial activities than CS but also show higher stability than natural phenolic compounds [16]. Till now, edible coating based on phenolic-g-CSs has been used to preserve different food items, such as silvery pomfret [15], peach [17], *Pleurotus eryngii* [18], mulberry [19], and meat [20]. Existing studies have demonstrated that edible coating based on phenolic-grafted-CSs shows a better food preservation effect than CS coating incorporated with free phenolic compounds [15,19].

Hydroxycinnamic acids belong to phenolic acids that possess potent antioxidant and antimicrobial activities. In our previous study, three different hydroxycinnamic acids including *p*-coumaric acid, caffeic acid and ferulic acid were individually grafted onto CS backbone through carbodiimide-mediated coupling reaction [21]. Among three hydroxycinnamic acid-g-CSs, ferulic acid-g-CS showed a good physical appearance and ideal antioxidant and antimicrobial activities, which is suitable to be used as an edible coating material for pork preservation. However, to our knowledge, no study has investigated the impact of ferulic acid-g-CS-based edible coating on the preservation of fresh pork. Therefore, this study aimed to evaluate the effect of ferulic acid-g-CS-based edible coating on the quality of pork during refrigerated storage.

## 2. Materials and Methods

### 2.1. Materials and Reagents

CS (deacetylated degree: 90%; average molecular weight: 1.5 × 10^5^ Da), ferulic acid, 1-ethyl-3-(3-dimethylaminopropyl) carbodiimide hydrochloride (EDC) and *N*-hydroxysuccinimide (NHS) and 1,1,3,3-tetramethoxypropane were purchased from Macklin Biochemical Co., Ltd. (Shanghai, China). All other reagents were of analytical grade.

### 2.2. Preparation of Ferulic Acid-g-CS Coating Solution

Ferulic acid-g-CS was prepared by EDC/NHS coupling reaction according to the previous study [21]. The grafting ratio of ferulic acid-g-CS was 91.75 mg/g based on Folin–Ciocalteu assay. To prepare ferulic acid-g-CS coating solution, 10 g of ferulic acid-g-CS was completely dissolved in 500 mL of 1% acetic acid aqueous solution (*v*/*v*) with constant stirring for 8 h at room temperature. Similarly, 2 wt% of CS coating solution was prepared in the same way. The pH of all coating solutions was adjusted to 5.6 ± 0.1 by sodium bicarbonate.

### 2.3. Pork Preparation and Coating

Fresh pork loins, the psoas major muscle along the central spine portion and ventral to the lumbar vertebrae, were bought from a local butcher (Yangzhou, China) at 24 h post-mortem. The pork loins were trimmed to remove visible connective tissue and fat and then cut into 2-cm-thick slices (10 cm × 5 cm). Afterwards, pork slices were randomly divided into three treatment groups: control group (samples without coating), CS coating group (samples coated with CS solution) and ferulic acid-g-CS coating group (samples coated with ferulic acid-g-CS solution). As for the coating treatment, pork slices were soaked in coating solutions for 30 s and then air-dried on stainless steel shelves at room temperature for 5 min to remove excessive coating solution on the surface of pork slices. The coating and dry treatments were repeated twice. All pork slices were packaged in aseptic polyvinyl chloride pallets and were sealed by polyethylene film. The pork slices were stored in a simulated shelf-life refrigerator with LED lighting. Three replicates of pork samples were collected and analyzed from each treatment group on each day. The coating layer on the pork surface was carefully removed before the quality measurement of the pork sample.

### 2.4. Determination of Total Viable Counts (TVC)

The TVC of pork sample was determined to evaluate microbial growth in the pork [22]. Briefly, 25 g of pork sample was homogenized with 225 mL of 0.9% sterile physiological saline in a sterile airtight bag for 2 min. Then, the homogenate was diluted with 0.9% sterile physiological saline by ten-fold serials. Subsequently, 0.1 mL of each diluted solution was distributed on plate count agar with incubating at 37 °C for 48 h. 

### 2.5. Determination of Total Volatile Basic Nitrogen (TVB-N)

The TVB-N value of pork sample was measured by Kjeldahl method [23]. Briefly, 10 g of pork sample was homogenized with 50 mL of distilled water at 10,000 rpm for 1 min. The homogenate was filtered, and 5 mL of filtrate was mixed with 5 mL of 10 g/L MgO solution, which was followed by distillation using the Kjeldahl distillation equipment (Jinan Hanon Instruments Co., Ltd., Jinan, China) for 5 min. Subsequently, the collected distillate was mixed with 10 mL of 20 g/L boric acid containing 5 drops of 0.1% methyl red and 0.1% bromocresol green. Finally, the obtained solution was titrated with 0.01 mol/L of hydrochloric acid. 

### 2.6. pH Measurement

Pork sample (10 g) was homogenized with 100 mL of distill water at 10,000 rpm for 1 min. Afterwards, the pH of the homogenate was determined by Mettler Toledo FE28 pH meter (Mettler Toledo International Inc., Shanghai, China) with temperature compensation [24]. The pH meter was calibrated beforehand by using standard buffers with pH value of 4.01 and 7.00.

### 2.7. Determination of Lipid Oxidation

Thiobarbituric acid reactive substances (TBARS) assay was conducted to evaluate the level of lipid oxidation in the pork [23]. Briefly, 5 g of pork sample was homogenized with 50 mL of 7.5% trichloroacetic acid solution at 10,000 rpm for 1 min. The homogenate was filtered, and 5 mL of filtrate was reacted with 5 mL of 0.02 mol/L thiobarbituric acid at 90 °C for 30 min. The reaction solution was cooled to room temperature and measured at 532 nm by Lambda 35 UV-Vis spectrophotometer (PerkinElmer Ltd., Waltham, MA, USA). The TBARS value was calculated based on the standard curve of 1,1,3,3-tetramethoxypropane and expressed as mg malonaldehyde (MDA) equivalents per kg of pork.

### 2.8. Determination of Drip Loss

The drip loss of pork during storage was measured by the method of Zhao et al. [25]. The initial weight of pork sample was immediately recorded before being packed in the packaging pallet. On each sampling day, the pork sample was taken out from the packaging pallet. After removing surface moisture of pork sample by absorbing paper, pork sample was weighed again to obtain the final weight. The drip loss of pork sample was calculated based on the initial and final weights of pork sample.

### 2.9. Microstructure Analysis

The microstructure of pork sample was analyzed by scanning electron microscopy (SEM) [26]. First, pork sample was cut into small cubes (1 × 1 × 1 cm) and then fixed in 0.1 mol/L phosphate buffer solution (pH 7.4) containing 2.5% of glutaraldehyde overnight. The sample was then rinsed with 0.1 mol/L phosphate buffer solution (pH 7.4) sthree times and sequentially dehydrated in 30%, 50%, 70% and 90% ethanol aqueous solutions for 10 min, which was followed by dehydrating twice in 100% ethanol for 15 min. The obtained dehydrated pork sample was sputtered with gold and observed by Gemini 300 SEM (Carl Zeiss, Oberkochen, Germany) at the voltage of 5 kV and the magnification of 100×.

### 2.10. Sensory Evaluation

The sensory evaluation of pork sample was performed by using a 5-point descriptive scale [24]. The sensory evaluation panelists were composed of ten trained members from College of Food Science and Engineering, Yangzhou University. Sensory evaluation was carried out in individual chambers under controlled light, temperature and humidity. Pork samples from different treatment groups were individually offered to each panelist. Meanwhile, fresh pork was also offered to panelists in order to compare with the stored pork samples. The color, odor, and over acceptance of pork sample were scored by the panelists using 5-point scale (5 = excellent, 4 = good, 3 = acceptable, 2 = poor and 1 = very poor). A rejection of the pork sample was achieved when the sensory score of pork sample was lower than 3.

### 2.11. Statistical Analysis

All data were expressed as mean ± standard derivation (SD). Results were analyzed by SPSS 13.0 software (SPSS, Inc., Chicago, IL, USA) by one-way analysis of variance and Duncan’s multiple range test. Results were considered statistically different if *p* < 0.05.

## 3. Results

### 3.1. TVC

**Figure 1:**
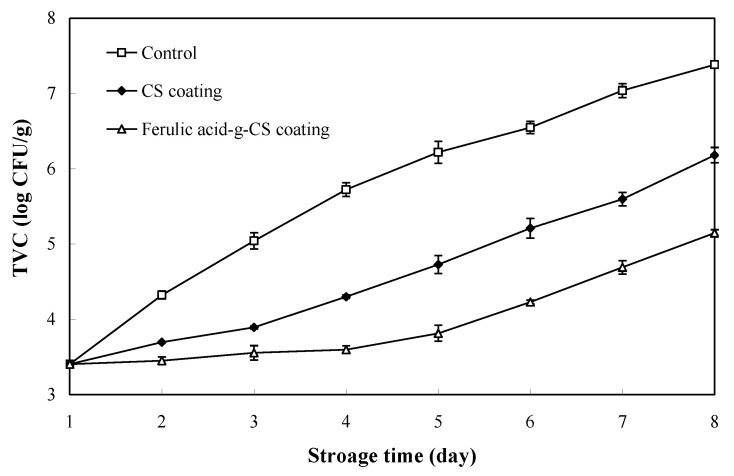
Changes in the total viable counts (TVC) of pork in the control group, CS) coating group and ferulic acid-g-CS coating group at 4 °C for 8 days. Data are presented as means ± SD of triplicates.

Changes in the TVC of pork during refrigerated storage are shown in Figure 1. The TVC value of pork in all the treatment groups showed gradually increased trends during storage. The increase in the TVC of pork during storage was related to the proliferation of psychrotrophic bacteria [11]. Pork in the control group showed the fastest growing rate of TVC. Chinese Standard GB/T 9959.2-2008 stipulates the TVC threshold of refrigerated pork is 6.00 log CFU/g meat. According to this standard, the TVC of pork in the control group and CS coating group exceeded the threshold at the 4th and 8th day, respectively. This was because CS had potential antimicrobial activity that could effectively inhibit microbial growth on the pork [9,24]. The antimicrobial mechanisms of CS are closely associated with the interactions between the positively charged amino groups of CS and the negatively charged microbial cell membrane, resulting in the breakdown of microbial cell membrane and leakage of intracellular substances [21]. Meanwhile, CS can form a barrier film around microbial cells, which effectively prevents the transport of nutrients into the cells [24]. Notably, the TVC value of pork in the ferulic acid-g-CS coating group was below 6 log CFU/g at the 8th day, indicating ferulic acid-g-CS coating had the highest antimicrobial activity. Yong et al. [21] recently demonstrated that the antimicrobial activity of CS was greatly improved by grafting with ferulic acid. The improved antimicrobial activity of ferulic acid-g-CS was mainly because the grafted ferulic acid moieties could disrupt microbial cell membranes and cause cytoplasmic leakage. Our results suggested that ferulic acid-g-CS coating was an effective way to reduce the TVC of pork during refrigerated storage. Zheng et al. [20] also found gallic acid-grafted-chitosan (gallic acid-g-chitosan) had stronger antimicrobial activity than CS, and gallic acid-grafted chitosan coatings effectively inhibited the increase of TVC during pork storage. Similarly, other researchers also found that CS coating incorporated with tarragon essential oils and gallic acid could effectively inhibit microbial growth on the pork [24,27]. 

### 3.2. TVB-N Value

**Figure 2:**
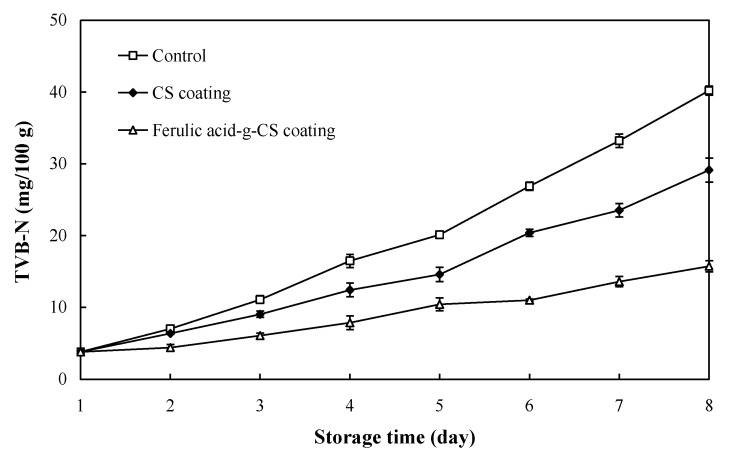
Changes in the TVB-N value of pork in the control group, CS coating group and ferulic acid-g-CS coating group at 4 °C for 8 days. Data are presented as means ± SD of triplicates.

TVB-N, mainly composed of trimethylamine, dimethylamine, and ammonia, is a parameter reflecting the spoilage degree of pork. The TVB-N limitation for fresh livestock products was 15 mg/100 g meat based on Chinese Standard GB 2707-2016. As shown in Figure 2, the TVB-N value of pork gradually increased during refrigerated storage, which was caused by the proliferation of spoilage bacteria that could degrade proteins in the pork, resulting in the breakage of muscle cell structures [25]. The destruction of muscle cell structures further led to the release of endogenous enzymes from pork tissues, which could accelerate protein degradation [23]. Notably, the TVB-N value of pork in the control group, CS coating group and ferulic acid-g-CS coating group exceeded the limitation of 15 mg/100 g at the 4th, 6th, and 8th day, respectively. As compared with the pork in the control group, pork in the CS and ferulic acid-g-CS coating groups showed significantly lower TVB-N values. This was because CS and ferulic acid-g-CS had antimicrobial activity that could retard the proliferation of spoilage bacteria as well as the degradation of proteins. Since ferulic acid-g-CS had stronger antimicrobial activity than CS [21], pork in the ferulic acid-g-CS coating group presented lower TVB-N values than pork in the CS coating group. Other researchers also found that gallic acid-g-CS coating [20], sodium alginate/carboxymethyl cellulose/epigallocatechin gallate coating [23] and CS/nisin/tea polyphenols coating [25] could effectively reduce the TVB-N level in the pork during refrigerated storage. 

### 3.3. pH Value

**Figure 3:**
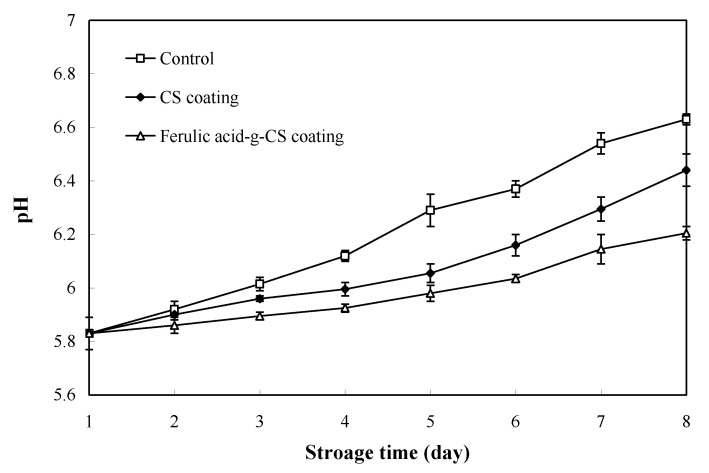
Changes in the pH value of pork in the control group, CS coating group and ferulic acid-g-CS coating group at 4 °C for 8 days. Data are presented as means ± SD of triplicates.

Changes in the pH value of pork during refrigerated storage are shown in Figure 3. The initial pH value of pork was 5.83, which was consistent with the results of Zhang et al. [28]. The pH value of pork in all the treatment groups continuously increased with the extension of storage time. The increase of pH value was related to the proliferation of spoilage bacteria that could degrade proteins and produce volatile bases [28]. As compared with the pork in the control group, pork in the CS and ferulic acid-g-CS coating groups showed relatively lower pH values during storage. At the 8th day, the pH value of pork in the control group, CS coating group and ferulic acid-g-CS coating group was 6.63, 6.44 and 6.21, respectively. Since CS and ferulic acid-g-CS coating both possess antimicrobial activity, they effectively inhibited the growth of spoilage bacteria and retarded the increase of pH in the pork. Ferulic acid-g-CS coating, due to its relatively higher antimicrobial activity than CS coating, was more effective in inhibiting microbial growth and pork spoilage. Other researchers also documented that gallic acid-g-CS coating [20], sodium alginate/carboxymethyl cellulose/epigallocatechin gallate coating [23], CS/essential oils coating [24] and CS/nisin/tea polyphenols coating [25] could also retard the increase of pH value during pork refrigerated storage.

### 3.4. TBARS Value

**Figure 4:**
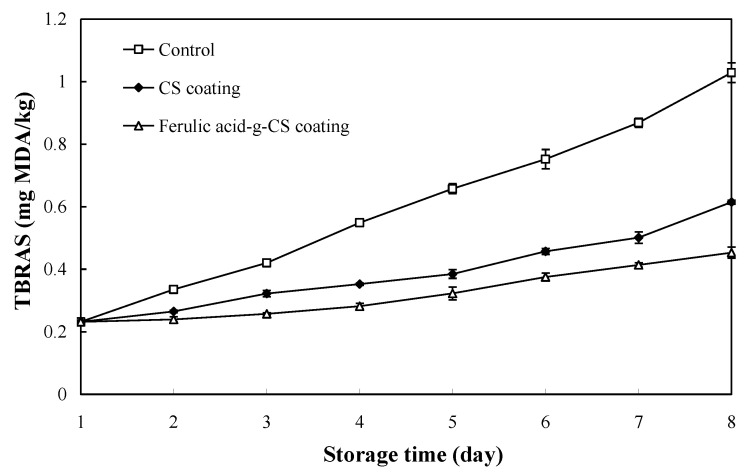
Changes in the TBARS value of pork in the control group, CS coating group and ferulic acid-g-CS coating group at 4 °C for 8 days. Data are presented as means ± SD of triplicates.

TBARS is a primary indicator reflecting the degree of lipid oxidation in the pork. As presented in Figure 4, the TBARS value of pork in all the treatment groups increased continuously during refrigerated storage. The TBARS value of pork in the control group dramatically increased from initial 0.23 mg MDA/kg to 1.03 mg MDA/kg at the 8th day. By contrast, the TBARS value of pork in the CS and ferulic acid-g-CS coating groups slightly increased to 0.61 and 0.45 mg MDA/kg, respectively, at the 8th day. The above results indicated CS and ferulic acid-g-CS coatings effectively lowered lipid oxidation degree during pork storage. This was because the pork in the control group was directly exposed to oxygen and was easily oxidized [11]. However, CS and ferulic acid-g-CS coatings could produce thin layer barriers outside the pork to retard oxygen permeation. Meanwhile, CS and ferulic acid-g-CS coatings possessed certain antioxidant activity, and thus could effectively retard the lipid oxidation of pork. It has been demonstrated that CS exerts antioxidant activity by interrupting free radical chain reaction via the formation of stable macromolecule radicals and the chelation of metal ions [29]. Yong et al. [21] further improved the antioxidant activity of CS by grafting with ferulic acid, which was because the abundant phenolic hydroxyl groups in the grafted ferulic acid moieties could effectively scavenge free radicals and chelate metal ions. In this study, pork in the ferulic acid-g-CS coating group presented lower TBARS values than pork in the CS coating group, which further demonstrated that ferulic acid-g-CS coating had higher antioxidant activity than CS coating. In other studies, researchers found gallic acid-g-CS coating [20], sodium alginate/carboxymethyl cellulose/epigallocatechin gallate coating [23], CS/essential oil coating [24], CS/nisin/tea polyphenols coating [25] and CS/gallic acid coating [27] had potentials to retard lipid oxidation of pork during refrigerated storage.

### 3.5. Drip Loss

**Figure 5:**
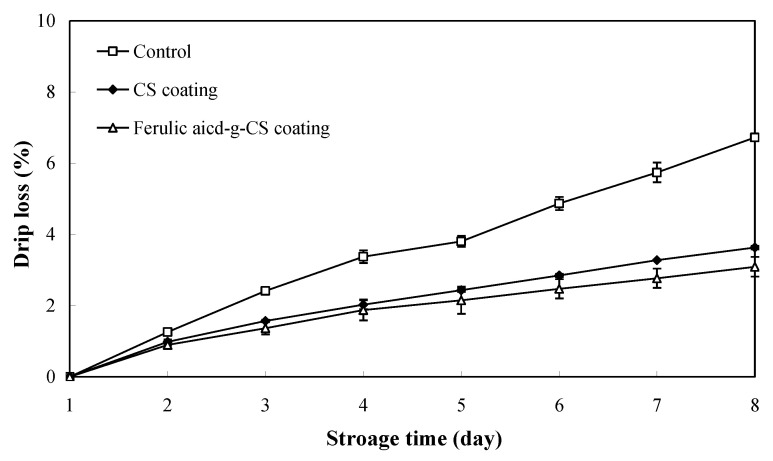
Changes in the drip loss of pork in the control group, CS coating group and ferulic acid-g-CS coating group at 4 °C for 8 days. Data are presented as means ± SD of triplicates.

Drip loss, a vital indicator reflecting the water holding capacity of edible coating, greatly influences the texture of pork. As shown in Figure 5, pork in the control group showed the highest drip loss, which increased to 6.72% at the 8th day. This was mainly because the uncoated pork was directly exposed to atmosphere and had a high moisture evaporation rate. By contrast, CS and ferulic acid-g-CS coatings effectively prevented the drip loss of pork due to the water holding capacity of coatings. On one hand, CS and ferulic acid-g-CS coatings could create semi-permeable barriers against moisture transfer, thereby limiting the moisture loss of pork [6]. On the other hand, CS and ferulic acid-g-CS contained abundant hydrophilic groups (e.g., hydroxyl and amino groups) in their structures, and thus exerted good water holding capacity. Notably, ferulic acid-g-CS coating was more effective in preventing the drip loss of pork in comparison with CS coating, indicating that ferulic acid-g-CS coating had a higher water holding capacity than CS coating. Yong et al. [21] demonstrated the hydrophilicity of CS was reduced by grafting with ferulic acid. As a result, ferulic acid-g-CS coating had a lower water vapor permeability and a denser structure than CS coating [21]. Moreover, the higher antioxidant and antimicrobial activities of ferulic acid-g-CS coating could retard the decomposition of muscle fibril and reduce the drip loss of pork [30]. Other researchers also reported that sodium alginate/carboxymethyl cellulose/epigallocatechin gallate coating [23] and CS/nisin/tea polyphenols coating [25] could effectively inhibit the drip loss of pork during refrigerated storage.

### 3.6. Microstructures

**Figure 6:**
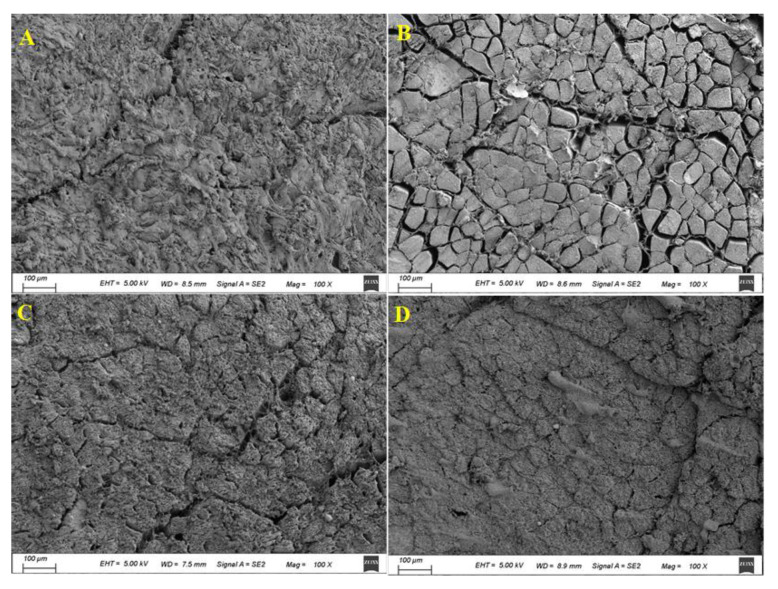
Microstructure of fresh pork (**A**) and pork stored in the control group (**B**), CS coating group (**C**) and ferulic acid-g-CS coating group (**D**) at 4 °C for 8 days.

The microstructures of fresh pork and the refrigerated pork stored in different treatment groups for 8 days are shown in Figure 6. Fresh pork presented a compact muscle structure with tight fibers. At the 8th day, pork in the control group showed a significantly less compact microstructure with some big gaps between muscle fibers, which indicated the dense structure and integrated muscle tissues of pork were seriously destructed during refrigerated storage. The micro-structural change of pork in the control group was mainly attributed to the drip loss of pork as well as the breakage of myofibrillar structure caused by the oxidation and microbial spoilage [11]. As compared with pork in the control group, pork in the CS and ferulic acid-g-CS coating groups showed remarkably denser microstructures at the 8th day. This suggested CS and ferulic acid-g-CS coatings could effectively maintain the texture of pork, which was because CS and ferulic acid-g-CS coatings were able to retard the drip loss of pork and inhibit the breakage of myofibrillar structure. Since ferulic acid-g-CS coating had higher antioxidant and antimicrobial activities than CS coating, pork in the ferulic acid-g-CS coating group showed a relatively denser microstructure without significant gaps between muscle fibers. The changes in the microstructure of refrigerated pork stored in different treatment groups were in agreement with the results of TVC, TVB-N, pH, TBARS. and drip loss.

### 3.7. Sensory Evaluation

**Figure 7:**
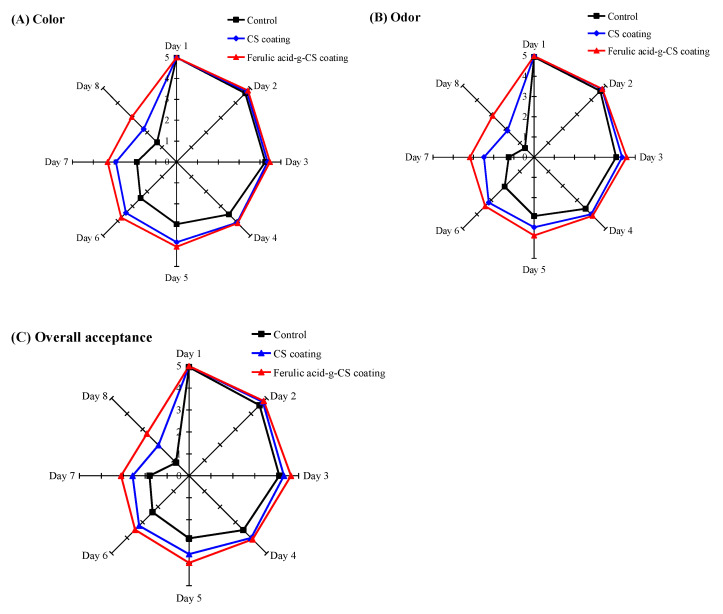
Changes in the sensory properties including color (**A**), odor (**B**), and over acceptance (**C**) of pork in the control group, CS coating group and ferulic acid-g-CS coating group at 4 °C for 8 days. Data are presented as means ± SD of triplicates.

Changes in the sensory attributes (e.g., color, odor, and over acceptance) of pork during refrigerated storage are shown in Figure 7. The color scores of pork in all the treatment groups gradually decreased during storage (Figure 7A) due to the oxidation of pork. Xiong et al. [11] suggested the deoxymyoglobin and oxymyoglobin pigments in pork were oxidized to form brown color pigment metmyoglobin, resulting in a reduction in redness. Pork in the control group gradually faded and showed an unacceptable color score (2.96) at the 5th day. By contrast, pork in the CS and ferulic acid-g-CS coating groups presented significantly higher color scores, indicating coating treatments effectively retard the oxidation of pork. Notably, pork in the ferulic acid-g-CS coating group had higher color scores than pork in the CS coating group, which was because ferulic acid-g-CS had higher antioxidant activity than CS. The red color of pork in the CS coating group significantly faded at the 7th day. By contrast, pork in the ferulic acid-g-CS coating group exhibited an acceptable red color even at the 8th day. 

The pork in the control group showed the lowest odor scores (Figure 7B), which was associated with pork spoilage [24]. CS and ferulic acid-g-CS coatings, due to their potential antioxidant and antimicrobial activities, effectively delayed the generation of off-odor in the pork. Pork in the CS and ferulic acid-g-CS coating groups exhibited unacceptable odor scores at the 7th and 8th day, respectively. This further confirmed ferulic acid-g-CS coating had relatively higher antioxidant and antimicrobial activities than CS coating. According to the overall acceptance score, pork in the control group, CS coating group and ferulic acid-g-CS coating group became unacceptable at the 5th, 7th, and 8th day, respectively. This suggested ferulic acid-g-CS coating obviously retarded the decrease in the overall acceptance of pork, which was attributed to the high antioxidant and antimicrobial activities of ferulic acid-g-CS coating. In other studies, researchers found the incorporation of antioxidant and antimicrobial substances (e.g., tomato plant extract [2], essential oils [24], and tea polyphenols [25]) into CS coating maintained the sensory attributes of pork. Recently, Şen and Kılıç [31] demonstrated that whey protein isolate-based coating incorporated with antioxidant matcha extract and antimicrobial acai powder extract had no negative effect on the overall acceptability of meatballs. In addition, Kakaei and Shahbazi [32] prepared antimicrobial films based on chitosan, gelatin, red grape seed extract, and *Ziziphora clinopodioides* essential oil, and found the films effectively maintained the sensory attributes of minced trout fillet. This study suggested active packaging films were also effective in maintaining the quality of meat products.

## 4. Conclusions

This study revealed that CS and ferulic acid-g-CS coatings had a positive influence on maintaining pork quality during refrigerated storage. Pork coated with CS and ferulic acid-g-CS significantly inhibited the microbial growth and lipid oxidation of pork during storage. Meanwhile, pork coated with CS and ferulic acid-g-CS showed better texture and sensory attributes than the uncoated pork during storage. Due to relatively higher antimicrobial and antioxidant activities, ferulic acid-g-CS coating was more effective in reducing microbial growth and lipid oxidation in the pork. The shelf life of refrigerated pork was extended to seven days by ferulic acid-g-CS coating. Results suggested ferulic acid-g-CS was a good coating material for pork preservation. In the future, the preservation effect of ferulic acid-g-CS coating on other meat products, such as chicken and beef, can be further evaluated. 

## Data Availability

The data presented in this study are available on request from the corresponding author.

## References

[1] Gavahian M., Chu Y.H., Jo C. (2019). Prospective applications of cold plasma for processing poultry products: Benefits, effects on quality attributes, and limitations. Compr. Rev. Food Sci. Food Saf..

[2] Chaparro-Hernández S., Ruiz-Cruz S., Márquez-Ríos E., Ornelas-Paz J.D.J., Del-Toro-Sánchez C.L., Gassos-Ortega L.E., Ocaño-Higuera V.M., López-Mata M.A., Devora-Isiordia G.E. (2019). Effect of chitosan-tomato plant extract edible coating on the quality, shelf life, and antioxidant capacity of pork during refrigerated storage. Coatings.

[3] Holman B.W., Kerry J.P., Hopkins D.L. (2018). Meat packaging solutions to current industry challenges: A review. Meat Sci..

[4] Hassan B., Chatha S.A.S., Hussain A.I., Zia K.M., Akhtar N. (2018). Recent advances on polysaccharides, lipids and protein based edible films and coatings: A review. Int. J. Biol. Macromol..

[5] Umaraw P., Munekata P.E., Verma A.K., Barba F.J., Singh V.P., Kumar P., Lorenzo J.M. (2020). Edible films/coating with tailored properties for active packaging of meat, fish and derived products. Trends Food Sci. Technol..

[6] Yong H., Liu J. (2021). Active packaging films and edible coatings based on polyphenol-rich propolis extract: A review. Compr. Rev. Food Sci. Food Saf..

[7] Yuan G., Chen X., Li D. (2016). Chitosan films and coatings containing essential oils: The antioxidant and antimicrobial activity, and application in food systems. Food Res. Int..

[8] Kumar S., Mukherjee A., Dutta J. (2020). Chitosan based nanocomposite films and coatings: Emerging antimicrobial food packaging alternatives. Trends Food Sci. Technol..

[9] Cao Y., Warner R.D., Fang Z. (2019). Effect of chitosan/nisin/gallic acid coating on preservation of pork loin in high oxygen modified atmosphere packaging. Food Control.

[10] Montaño-Sánchez E., Torres-Martínez B.D.M., Vargas-Sánchez R.D., Huerta-Leidenz N., Sánchez-Escalante A., Beriain M.J., Torrescano-Urrutia G.R. (2020). Effects of chitosan coating with green tea aqueous extract on lipid oxidation and microbial growth in pork chops during chilled storage. Foods.

[11] Xiong Y., Chen M., Warner R.D., Fang Z. (2020). Incorporating nisin and grape seed extract in chitosan-gelatine edible coating and its effect on cold storage of fresh pork. Food Control.

[12] Yaghoubi M., Ayaseh A., Alirezalu K., Nemati Z., Pateiro M., Lorenzo J.M. (2021). Effect of chitosan coating incorporated with *Artemisia fragrans* essential oil on fresh chicken meat during refrigerated storage. Polymers.

[13] Zhang H., Li X., Kang H. (2019). Chitosan coatings incorporated with free or nano-encapsulated *Paulownia Tomentosa* essential oil to improve shelf-life of ready-to-cook pork chops. LWT.

[14] Cianciosi D., Forbes-Hernández T.Y., Afrin S., Gasparrini M., Reboredo-Rodriguez P., Manna P.P., Zhang J., Lamas L.B., Flórez S.M., Toyos P.A. (2018). Phenolic compounds in honey and their associated health benefits: A review. Molecules.

[15] Wu C., Fu S., Xiang Y., Yuan C., Hu Y., Chen S., Liu D., Ye X. (2016). Effect of chitosan gallate coating on the quality maintenance of refrigerated (4 °C) silver pomfret (*Pampus argentus*). Food Bioprocess Technol..

[16] Liu J., Pu H., Liu S., Kan J., Jin C. (2017). Synthesis, characterization, bioactivity and potential application of phenolic acid grafted chitosan: A review. Carbohydr. Polym..

[17] Jiao W., Shu C., Li X., Cao J., Fan X., Jiang W. (2019). Preparation of a chitosan-chlorogenic acid conjugate and its application as edible coating in postharvest preservation of peach fruit. Postharvest Biol. Technol..

[18] Liu J., Meng C.G., Wang X.C., Chen Y., Kan J., Jin C.H. (2016). Effect of protocatechuic acid-grafted-chitosan coating on the postharvest quality of *Pleurotus eryngii*. J. Agric. Food Chem..

[19] Yang C., Han B., Zheng Y., Liu L., Li C., Sheng S., Zhang J., Wang J., Wu F. (2016). The quality changes of postharvest mulberry fruit treated by chitosan-g-caffeic acid during cold storage. J. Food Sci..

[20] Zheng M., Zhang C., Zhou Y., Lu Z., Zhao H., Bie X., Lu F. (2018). Preparation of gallic acid-grafted chitosan using recombinant bacterial laccase and its application in chilled meat preservation. Front. Microbiol..

[21] Yong H., Liu Y., Yun D., Zong S., Jin C., Liu J. (2021). Chitosan films functionalized with different hydroxycinnamic acids: Preparation, characterization and application for pork preservation. Foods.

[22] Wang C., Yang J., Zhu X., Lu Y., Xue Y., Lu Z. (2017). Effects of *Salmonella* bacteriophage, nisin and potassium sorbate and their combination on safety and shelf life of fresh chilled pork. Food Control.

[23] Ruan C., Zhang Y., Sun Y., Gao X., Xiong G., Liang J. (2019). Effect of sodium alginate and carboxymethyl cellulose edible coating with epigallocatechin gallate on quality and shelf life of fresh pork. Int. J. Biol. Macromol..

[24] Zhang H., Liang Y., Li X., Kang H. (2020). Effect of chitosan-gelatin coating containing nano-encapsulated tarragon essential oil on the preservation of pork slices. Meat Sci..

[25] Zhao S., Li N., Li Z., He H., Zhao Y., Zhu M., Wang Z., Kang Z., Ma H. (2019). Shelf life of fresh chilled pork as affected by antimicrobial intervention with nisin, tea polyphenols, chitosan, and their combination. Int. J. Food Prop..

[26] Cheng S., Wang X., Yang H., Lin R., Wang H., Tan M. (2020). Characterization of moisture migration of beef during refrigeration storage by low-field NMR and its relationship to beef quality. J. Sci. Food Agric..

[27] Fang Z., Lin D., Warner R.D., Ha M. (2018). Effect of gallic acid/chitosan coating on fresh pork quality in modified atmosphere packaging. Food Chem..

[28] Zhang H., He P., Kang H., Li X. (2018). Antioxidant and antimicrobial effects of edible coating based on chitosan and bamboo vinegar in ready to cook pork chops. LWT.

[29] Bi F., Yong H., Liu J., Zhang X., Shu Y., Liu J. (2020). Development and characterization of chitosan and D-α-tocopheryl polyethylene glycol 1000 succinate composite films containing different flavones. Food Packag. Shelf Life.

[30] Traore S., Aubry L., Gatellier P., Przybylski W., Jaworska D., Kajak-Siemaszko K., Santé-Lhoutellier V. (2012). Higher drip loss is associated with protein oxidation. Meat Sci..

[31] Şen D.B., Kılıç B. (2021). Effects of edible coatings containing acai powder and matcha extracts on shelf life and quality parameters of cooked meatballs. Meat Sci..

[32] Kakaei S., Shahbazi Y. (2016). Effect of chitosan-gelatin film incorporated with ethanolic red grape seed extract and *Ziziphora clinopodioides* essential oil on survival of *Listeria monocytogenes* and chemical, microbial and sensory properties of minced trout fillet. LWT-Food Sci. Technol..

